# Exploring work stressors and resources and their perceived effects on symptoms and work ability in employees with multiple sclerosis: a qualitative study

**DOI:** 10.1186/s12883-026-05417-z

**Published:** 2026-09-24

**Authors:** Jessica T. Bau, Lisa Guthardt, Jennifer Apolinário-Hagen, Adrian Loerbroks, Sven G. Meuth, Jeannette Weber

**Affiliations:** 1 Institute of Occupational, Social and Environmental Medicine, Centre for Health and Society, Medical Faculty, University Hospital Düsseldorf, Heinrich Heine University Düsseldorf, Düsseldorf, Germany; 2 Institute of Medical Sociology, Centre for Health and Society, Medical Faculty, University Hospital Düsseldorf, Heinrich Heine University Düsseldorf, Düsseldorf, Germany; 3 Institute of Family Medicine/General Practice, Primary Care Research Group, Medical Faculty, University of Duisburg-Essen, Essen, Germany; 4 Department of Neurology, Medical Faculty and University Hospital, Heinrich Heine University Düsseldorf, Düsseldorf, Germany; 5 Düsseldorf, Germany

**Keywords:** Multiple sclerosis, Occupational stress, Health resources, Working conditions, Symptoms, Work ability, Qualitative research

## Abstract

**Background:**

Adverse working conditions can lead to reduced working hours, career changes, or early exit from the workforce among employees with multiple sclerosis. The aim of the present study was to explore work-related stressors and resources, as well as their effects on symptoms and work ability, as perceived by employees with multiple sclerosis in Germany.

**Methods:**

Semi-structured interviews were conducted via video conference and telephone. The interviews were recorded, transcribed, and analyzed using MAXQDA and qualitative content analysis according to Kuckartz.

**Results:**

Of the 26 participants (73.1% women, mean age 38.4 years, SD = 10.2), 73.1% worked full-time and 26.9% part-time (> 19 h) across various sectors, including, e.g., public services, healthcare, IT, trade and industry, and crafts. Stressors and resources were categorized into three groups: (1) work-related factors (work contents and demands, work organization, and workplace conditions), (2) social factors (relationship with supervisors and colleagues), and (3) person-related factors (self-expectations, attitudes towards work and handling of the disease). Reported work stressors contributed to symptoms, such as fatigue, sensory disturbances, and cognitive and visual impairments, which impacted work ability. Reported work resources enhanced cognition and fine motor skills, distracted participants from focusing on their disease, and fostered motivation and energy to carry out their profession.

**Conclusions:**

The results provide insights into which factors are essential for improving well-being and work ability in employees with multiple sclerosis. Based on these findings, workplace interventions and adaptations can be considered to enable employees with multiple sclerosis to remain in the workforce for a longer time.

**Clinical trial number:**

Not applicable.

**Supplementary Information:**

The online version contains supplementary material available at https://doi.org/10.1186/s12883-026-05417-z.

## Background

Multiple sclerosis (MS), a chronic disease of the central nervous system, is one of the most frequent neurologic conditions among younger adults [1, 2]. Common symptoms can be visual, sensory and motor impairments, pain, fatigue and cognitive decline [3]. The diagnosis is often made between the ages of 20 and 40 [4], thus in a phase of potentially high professional productivity.

Employees with MS (ewMS) are at higher risk of reducing their working hours, changing or quitting their job and retiring early [5, 6]. Up to 80% of ewMS stop working within 15 years after having received the diagnosis [7], which can have a negative impact on their financial security, social integration and self-esteem [8]. It can be assumed that ewMS can remain longer in paid work when work demands correspond to individual work capacity [9] and when sufficient resources (e.g., social support or control) are available [10]. According to the Job-Demands-Resources-Model, an imbalance between job demands (i.e., physical, psychological, organizational or social aspects) and resources to meet those demands (such as a good relation to the supervisor, performance feedback or autonomy) can result in strain and work difficulties [11, 12].

Potentially harmful work-related factors for ewMS identified in prior research include the temperature at the workplace, limited accessibility (e.g., too many stairs) [13], a lack of information about work regulations or potential jobs for people with disabilities [14], commuting [15], negative reactions [16] or a negative attitude of employers and colleagues (e.g., lack of understanding, especially with invisible symptoms) [15]. Other factors include stigma [17], discrimination [16], MS symptoms that cause work stress (e.g., walking impairments, fatigue, cognitive problems) [2] or worries about the future due to the uncertain MS progression [18]. Resources and adjustments from the research literature cover adaptions to telecommuting and work flexibility, the possibility to work from home and to customize the job according to one’s individual abilities [16], social support from managers, colleagues and networks [7], accessibility of parking [14] or preferred parking and ergonomic workplaces [19], autonomy and control [10] as well as individual modifications or strategies (e.g., recording notes via smartphone, writing things down, making use of technological devices and aids) [16, 20]. A previous study has already found that adjustments at the workplace as well as symptom management can be beneficial for employees with MS [21]. Earlier research indicated the importance of setting priorities, planning ahead, making decisions and adaptations to the workday or to work tasks [20]. In this context, awareness for symptoms/self-monitoring and being able to react accordingly to change current work strategies, thus listening to your own body and health, was mentioned as important. Another qualitative study from Germany among people with MS with lifestyle-adjustments (i.e., nutrition, physical activity and stress management) that was not conducted in the context of work explored potential positive effects of physical activity and good nutrition and found that individuals with MS sometimes had difficulties implementing these in their daily lives [22].

However, several previous studies follow a quantitative approach, and study populations vary and often include not only ewMS but also people with MS who were volunteers (i.e., working on a voluntary basis), on disability pension, unemployed or in (early) retirement (e.g [16, 18, 20]). Others set a broader or more narrowed focus than the present study, including e.g., people with MS, employers and healthcare professionals in one study [15], or only a subpopulation like women with MS [7], and no previous study encompassed the perceived effects of resources and stressors on symptoms and work ability to the best of our knowledge. Additionally and despite the call for country-specific knowledge on MS [23], a systematic review on work barriers and job adjustments of ewMS showed that there are studies from, e.g., Australia, the USA, the UK, Spain, France, the Netherlands and Switzerland but none for Germany [14].

Therefore, we conducted a study that addresses both work stressors and resources in relation to perceived effects on MS symptoms and work ability in one go and chose the study population of on-the-job employees in Germany. We used a qualitative approach to gain profound insights into the interplay of stressors, resources, symptoms and work ability among ewMS from various sectors in Germany, as qualitative research can add further context, more in-depth insights and understanding to quantitative data [24].

We pursued the following research questions (RQ):

- RQ1: Which stressors do ewMS experience at work?
- RQ2: How are those stressors perceived to affect MS symptoms and work ability according to ewMS?
- RQ3: Which occupational resources do ewMS experience at work?
- RQ4: How do those resources affect MS symptoms and work ability according to ewMS?

## Methods

We conducted semi-structured interviews to identify work stressors and resources as well as their impact on symptoms and work ability experienced by ewMS. The interviews were analyzed using content analysis, drawing on Kuckartz [25]. Our reporting followed the Consolidated Criteria for Reporting Qualitative Research (COREQ [26], see Additional file 1).

### Study team

The study team consisted of the following researchers: Jessica T. Bau (JTB, doctoral candidate in public health, master’s degree in social science [27, 28]), Lisa Guthardt (LG, research associate, master’s degree in Literary Translation [29, 30]), Jennifer Apolinário-Hagen (JAH, qualified psychologist, senior researcher [31, 32]), Adrian Loerbroks (AL, health scientist/epidemiologist and occupational health senior researcher, expertise in health–work relationships in chronic disease [33, 34]), Sven G. Meuth (SGM, neurologist treating MS patients for 20 years [35, 36]) and Jeannette Weber (JW, senior researcher, degrees in Biomedical Sciences and Public Health, experience in occupational health research [37, 38]). All researchers except for SGM also had expertise in qualitative research.

### Participants and recruiting

Individuals were eligible to participate if they were currently employed for at least 19 h per week (to collect data from regularly employed individuals who work at least 50% part-time), aged between 18 and 67 years and reported to be physician-diagnosed with MS for at least one year. Exclusion criterion was a recent MS relapse (within the last six months). Sufficient German language skills were required to participate in the interview.

The recruitment material consisted of a digital flyer and a website with information about the project on the homepage of the Institute of Occupational, Social and Environmental Medicine of the Heinrich Heine University Düsseldorf, Germany. Both the flyer and the website contained brief information about the study background and its objectives as well as inclusion and exclusion criteria. Additionally, we gave information on an expense allowance of 15 euros and offered three ways to contact us for participation. Potential participants could sign up via a link or QR code, by phone or e-mail. Recruitment material was shared via MS self-help groups on Instagram and Facebook (Germany-wide recruitment), the mailing lists of a non-profit entrepreneurial company that offers a platform for exchange among individuals affected by MS, and several self-help groups (local recruitment in the area of Düsseldorf). The flyer was also distributed in print in the outpatient clinic of the Department of Neurology at the University Hospital Düsseldorf. Participants were also recruited via personal contacts of the researchers or personal contacts of study participants.

We initially recruited participants using convenience sampling. After 20 interviews, we applied purposive/criterion sampling, targeting age groups and professional backgrounds that had not yet been represented (i.e., individuals in their 40s and blue-collar workers), to maximize the breadth of perspectives and opinions captured. This was possible because many more potential participants volunteered than were needed after advertising our study on Instagram.

Apart from one person, all participants used the link or QR code to enter their contact information (name, e-mail, telephone number) online via SoSci Survey (SoSci Survey GmbH, Munich, Germany; web application for creating online questionnaires). Registration was open from November 8 to December 7, 2023. In this period, 86 individuals registered as potential participants. Of these, 35 were contacted by e-mail to make an appointment for the interview. In general, participants were selected in chronological order, according to the receipt of their e-mail. As described above, the last participants were intentionally selected based on age and professional background. They were then sent personalized links with individual participant codes (for pseudonymization) and were asked to carefully read the study information, provide informed consent (click to agree) and answer a questionnaire on sociodemographic data (age, gender, highest level of education), their occupation (profession, working hours) and disease (e.g., diagnosis, course, last relapse, degree of disability). The background questionnaire can be found in the additional files [see Additional file 2]. In total, 26 participants provided informed consent and took part in the interview. Ethical approval (approval number 2023–2318) was obtained from the ethics committee of the Medical Faculty of the Heinrich Heine University Düsseldorf before the collection of data.

### Interview guide and data collection

The initial interview guide was developed by JW based on the identified research gap and research question, which were previously derived from the review of the existing literature. There were brainstorming and refinement sessions (first with JAH and LG, then with JAH, LG, AL and JTB) to further discuss relevant issues and the adequate formulation of questions. Before the beginning of the recruitment, the guide was also discussed with SGM, who has experience in treating MS patients for 20 years. The guide was slightly modified again after two pretest interviews with two personal contacts also affected by MS before the official recruitment. The final version of the interview guide can be found in the additional files [see Additional file 3]. Both the background questionnaire and the interview guide were translated from German into English for the purpose of publication. The interview guide covered background information on the respondent’s typical workday, work history and medical history, including the respondent’s current therapy (such as medication) and responsibilities outside of work like childcare. The focus, however, was on perceived stressors and resources at work, as well as perceived effects on symptoms and work ability. Specifically, participants were asked: “Are there factors in your work that cause you stress?” and “What do you like about your job? Are there any factors that benefit you?” Next, they were asked: “To what extent do you believe that the factors you just mentioned affect your MS disease?”. If not mentioned, participants were specifically asked about symptoms and work ability. In addition, the interviews addressed reasons for and against disclosure of the disease at work as well as positive and negative consequences. Due to the different foci of these questions, results will be reported elsewhere.

The majority of the semi-structured interviews were conducted online using the open-source video conference software BigBlueButton (BigBlueButton Inc., Ottawa, Canada). The telephone was used when technical difficulties occurred, or at the request of the participants. The interviewer conducted the interviews from an office or home office, with no other individuals present in the room, while participants joined from their own homes. Discussing the disease and MS-related difficulties at work may potentially cause emotional distress for participants. Therefore, at the beginning of each interview, participants were asked how they were feeling that day, and all were in sufficiently good condition to take part. In addition, all interviews were conducted while participants were at home, in a familiar and comfortable environment. All interviews were audio-recorded with a digital recording device by JTB between November and December 2023. Interviews were conducted until thematic saturation was likely to be reached, i.e., the point where no additional data seems to emerge [39]. Data saturation was monitored continuously during data collection and analysis. After 23 interviews, we observed thematic redundancy. The last three interviews were used to confirm this saturation, during which only few additional subcodes were identified but no new themes emerged. Consequently, recruitment was stopped at 26 participants. The verbatim transcription of the interviews according to Dresing & Pehl [40] was subsequently performed by an external service provider that adheres to German data security standards. After transcription, the data was anonymized, and audio records were deleted.

### Data analysis

All interviews were analyzed using qualitative content analysis according to Kuckartz [25] with the software MAXQDA 2024 (VERBI Software, Berlin, Germany). We employed a deductive-inductive approach, with main categories based on the interview guide and subcodes emerging inductively from the data. We used an iterative, team-based approach to develop and refine the coding system: JTB and LG independently coded the first three interviews, compared and discussed their codes to agree upon a preliminary version of the code system. After coding five additional interviews, discrepancies were reviewed and resolved through discussion, resulting in an adapted version of the code system. This process was repeated for five more interviews, after which JTB discussed the code system with JW, leading to minor adjustments. Using the finalized coding system, JTB revised the previously coded interviews once again and coded the second half of the data. After all data had been coded, LG reviewed all coded material to ensure alignment with the coding system. Any remaining issues or ambiguities were resolved through discussion with JW. Participants did not review transcripts or preliminary findings.

Interviewer reflexivity was addressed by having the interviewer follow a guide developed collaboratively by the research team. Any instances where interviewer influence on responses was suspected were documented in reflective memos and discussed by the team.

## Results

### Participants’ characteristics

Twenty-six interviews (20 online via video conference, 6 by phone) were conducted with an average duration of 40.6 min (range: 28.24–60.59 min). Sample characteristics are shown in Table 1. Participants were between 26 and 59 years old with an average of 38.4 years (standard deviation (SD): 10.2). 73.1% of interviewees were female. The respondents reported that, on average, around six years (SD: 8.5) had passed since their diagnosis, with some individuals diagnosed just one year ago, and others up to 40 years ago. We succeeded in including participants from different sectors and industries: public services (e.g., scientist, teacher, clerk), healthcare (e.g., doctor, nurse, social worker), IT (e.g., software developer), trade and industry (e.g., marketing clerk, food quality assurance specialist), law (legal trainee), crafts (carpenter), construction (risk manager) and church (parish assistant). Additional file 4 provides the assignment of participants to their respective occupations, along with information on work hours.

**Table 1:** Sample characteristics (n=26)

Characteristics		
Age (mean (SD^a^; min-max))		38.42 (10.2; 26–59)
Female, n (%)		19 (73.08)
Highest level of education, n (%)	No school-leaving qualification	0 (0.00)
	German ‘Haupt-/Volksschulabschluss’ (i.e., lower secondary school-leaving certificate)	0 (0.00)
	German ‘Mittlere Reife/Realschulabschluss‘ (i.e., intermediate secondary school-leaving certificate)	2 (6.47)
	German ‘Fachhochschulreife’ (i.e., advanced technical college entrance qualification)	1 (2.88)
	German ‘Abitur/allgemeine Hochschulreife‘ (i.e., general qualification for university entrance)	6 (21.58)
	University education (bachelor’s/master’s degree or higher)	18 (69.06)
Employed full-time, n (%)		19 (73.08)
Years since diagnosis (mean (SD; min-max)		6.16 (8.51; 1–40)
Course of disease, n (%)	Clinically isolated syndrome	0 (0.00)
	Relapsing-remitting	22 (84.62)
	Secondary progressive	3 (11.54)
	Primary progressive	0 (0.00)
	Not indicated	1 (3.85)
Able to walk > 500 m, n (%)^b^		23 (88.46)
Degree of disability^c^, n (%)		13 (50.00)

^a^SD = Standard deviation

^b^Referring to the self-reported disability status scale [41]

^c^Self-reported statements regarding the officially determined German “Grad der Behinderung" (English: degree of disability), which can provide legal benefits, such as tax advantages, additional vacation rates or entitlement to care and health services

### Main results

**Fig. 1:**
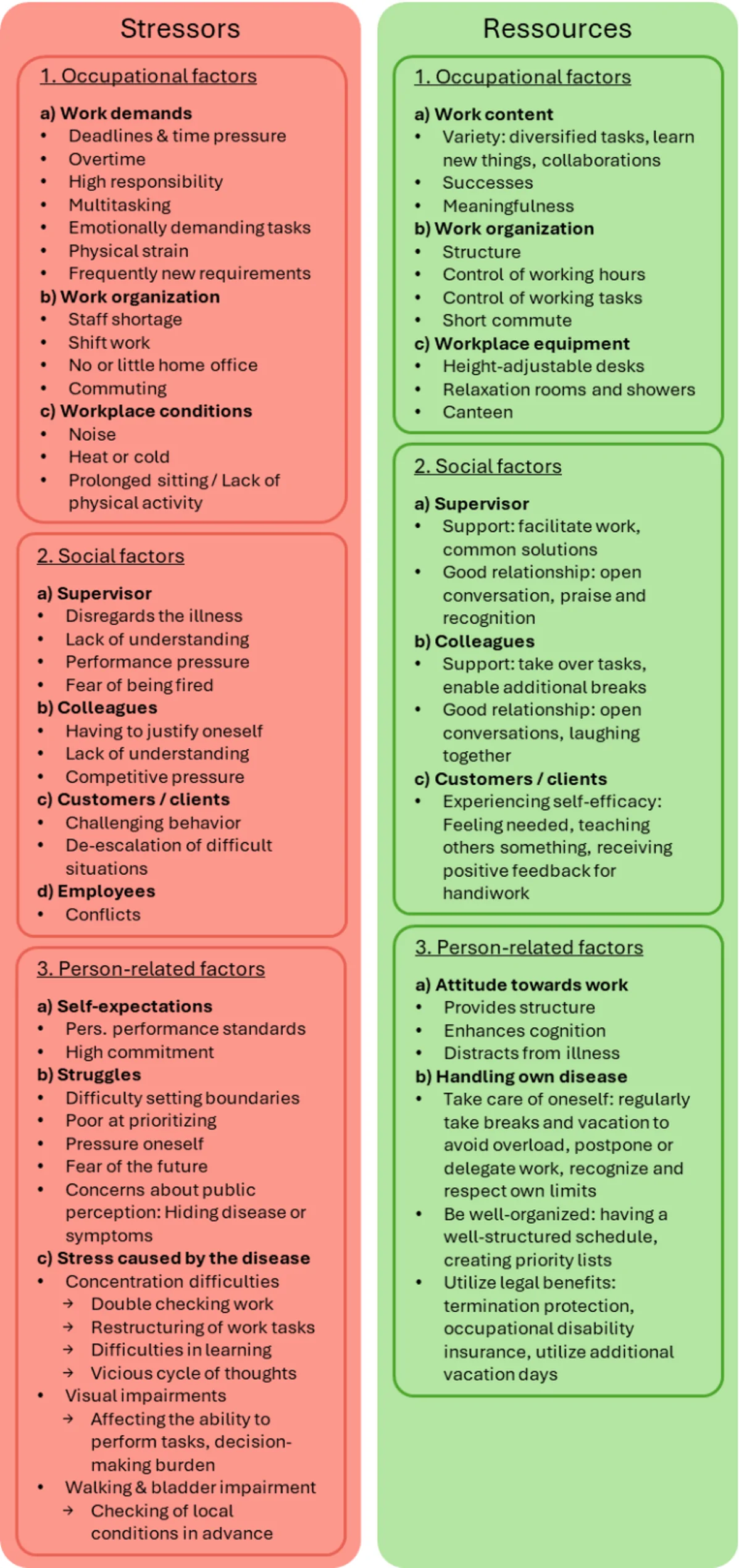
Coding System: First and second level codes of stressors and resources mentioned by participants

Figure 1 provides an overview of the most important identified stressors and resources, which were divided into three categories, respectively: (1) occupational factors (referring to work demands, work organization and workplace equipment), (2) social factors (relationship with supervisors and colleagues) and (3) person-related factors (self-expectations, struggles and disease-related stressors, attitudes towards work and handling the disease).

### Work stressors and their perceived effect on symptoms and work ability

It was not always possible to attribute specific effects to individual stressors (since there was no separate inquiry after each individually mentioned stressor). Sometimes, the description of the effects on symptoms and work ability seemed to refer to the cumulative experience of work stress. The relationship between stressors and their impact on symptoms and work ability is described in the following chapters. Due to limited space not all subcategories can be elaborated on.

#### Occupational factors

##### a) Work demands

EwMS reported typical stressors related to work demands, such as deadlines, time pressure or overtime. As stress reactions, interviewees frequently reported tingling in the limbs or numbness in different body parts. Participants indicated that they had experienced certain sensory disturbances at the onset of the disease, which then reappeared during periods of stress. These symptoms were often reported to be short-lived, but an indication to slow down, relax or get some sleep. Fatigue was the most frequently reported symptom in combination with occupational stress. Some noted that the fatigue accumulated and worsened as the week progressed. Consequently, they reported the need to recover on evenings and the weekend, which inevitably reduced their time for leisure activities.

*“I have to say*,* there are days when I come home and the fatigue kicks in right away. I don’t know if you’re familiar with the issue of fatigue? Your body suddenly becomes incredibly tired and is of no use anymore. And this happens more often when there’s been stress at work*,* or when things haven’t gone well.” (P10)*.

##### b) Work organization

Participants reported stressors such as staff shortage and complained about adverse working time regulations like shift work as well as a lack or an insufficient extension of options to work from home. A rigid regulation of working hours and office days made it difficult for the respondents to take breaks or lie down in an instant during the day, if necessary, or attend regular medical appointments:

*“It’s definitely relieving to have the opportunity to work from home*,* but we also have certain rules that really stress me out. We have fixed office days*,* and you can’t change them at all*,* so you have to be there. And that’s obviously difficult for me. Those are Tuesdays and Thursdays*,* and these are also the days when doctors have longer consultation hours.” (P7)*.

##### c) Workplace conditions

Regarding workplace conditions (equipment and environment), participants complained about a lack of physical activity (especially white-collar workers) and high noise levels (e.g., in hospitals and schools). Most frequently, they reported having to deal with high temperatures in the summer due to a lack of air conditioning at the workplace. They talked about Uhthoff’s phenomenon and explained that heat leads to a worsening of MS symptoms caused by an increase in body temperature, making work increasingly difficult. In this context, they also mentioned specific symptoms such as physical weakness as well as cognitive (e.g., concentration difficulties, forgetfulness or word-finding problems) and visual impairments:

*“Well*,* when it’s hot*,* my vision definitely gets worse. And I also notice that […] this is accompanied by a greater physical weakness. Like*,* my movements slow down*,* my physical performance is significantly reduced overall. […] You can really tell that the brain isn’t working properly*,* normal thought processes are just not working. And then you sometimes feel*,* so to speak*,* like you can’t even put two and two together.” (P11)*.

#### Social factors

##### a) Supervisor and b) Colleagues

Some participants reported insufficient understanding and consideration of the disease by their superiors. This included inadequate assessment of tasks given their MS, leading to the need for repeated explanations. Additionally, some interviewees expressed fear of job loss due to performance pressure exerted by their supervisors:

*“And I also talked to my supervisor about it in good time. And*,* yeah*,* she made an effort*,* she was also empathetic*,* but the conversation just put me under pressure after all. Because she was very clear with me: ‘I know you’re sick*,* I get it*,* it’s really bad*,* but if you want to keep this position*,* I still expect top performance. So*,* you can’t slack off*,* you can’t perform less. We need to keep an eye on your sick days.” (P7)*.

The relationship with colleagues was rarely described as difficult. If so, respondents felt the need to justify themselves, e.g., when they had to decline a task or request to switch tasks with a colleague due to their (invisible) symptoms. Few respondents additionally mentioned competitive pressure among colleagues (i.e., elbow mentality, competition for who performs best).

In connection with conflictual social interactions or a perceived overload when many people simultaneously demanded something, participants reported lifting their feet became increasingly difficult, for example, when climbing stairs or getting into the car to drive home.

Occasionally, participants reported feeling patronized by colleagues when they judged whether a work task might be too stressful for the affected person and recommended taking it easy, even though the person felt capable of handling it.

#### Person-related factors

##### a) Self-expectations and b) Struggles

Some respondents mentioned stress-reinforcing self-expectations such as high personal performance standards. They put themselves under pressure to work as well and as long as possible out of fear that the disease might progress and they would no longer be able to work:

*“Soon*,* your mind is racing and you start thinking: I need to get this done quickly before I may be feeling worse or something. It’s like when you realize you’re about to catch a cold*,* you’re about to get sick. And then you also want to get everything done*,* so you push through one more time.” (P16)*.

Some also talked about the stress-inducing feeling of not doing enough and feelings of guilt when they took a break or missed work due to symptoms or a medical appointment. They reported comparing themselves with healthy colleagues, which seemed to create a feeling of wanting to or having to keep up. According to them, this feeling intensified when performance pressure was high within the company.

Some participants indicated adverse predispositions that created or intensified stress, such as problems with structuring and prioritizing their tasks as well as general difficulties in dealing with stressful situations. They described having difficulties maintaining their own boundaries and keeping their limits so that they tended to work too much.

##### c) Stress caused by the disease

Interviewees reported that stress at work was also directly caused by MS symptoms. People with walking and/or bladder impairments stated that appointments outside their regular workplace needed careful advance planning (e.g., meetings with external business partners):

*“At first*,* I’m always a bit hesitant and I look around: How big is this building? And where do I have to go? Are there a lot of stairs*,* or is there an elevator? […] Where are the restrooms? These are basically some stressors*,* that I need to have a look first to find out how to navigate the place. […] And that already concerns me and it stresses me out in advance.” (P17)*.

Several participants talked about a perceived burden to decide whether they were still able to perform tasks or work at all. That was perceived as especially high when motoric or visual impairments posed risks to others (e.g., in healthcare) or to oneself (e.g., in crafts).

*“Well*,* of course*,* if there’s something that doesn’t work in terms of fine motor skills*,* then I have to find a colleague or whatever. But I’m also open and honest about it*,* and I’ll tell them*,* ‘Sorry*,* I can’t do that today.’ And I don’t go into the shock rooms [explanation: special unit in an emergency department to take care of seriously ill or severed patients] when I don’t feel capable of doing that. Sometimes*,* these decisions need to be made in a split second*,* when there’s not much time.” (P19)*.

Some respondents stated that the perception of (mild) symptoms could lead to a vicious cycle where focusing on the symptoms resulted in a perceived worsening of the condition and thus further distraction from work.

#### Key consequences of work stressors

Generally, respondents described different effects of work stress on perceived symptoms and work ability. Participants reported sensory disturbances (e.g., tingling or numbness) as an indicator of high job strain and cognitive impairments (e.g., concentration difficulties) as a consequence of high temperatures at the workplace. In connection with conflictual social interactions and high work demands, participants reported walking impairments, stiff and cramped or wobbly legs, fatigue and reduced physical strength.

### Work resources and perceived effects on symptoms and work ability

#### Occupational factors

##### a) Work content and b) Work organization

Participants found their work enriching and pleasant when it was characterized by varying daily schedules, versatile tasks and contact with different professions and nationalities, preventing boredom and monotony. They valued training courses, new input and ideas, which made them develop new skills and seemed to have a positive effect on cognition:

*“It’s just the cognitive aspect*,* because if you don’t have to use your brain*,* there’s not much going on up there eventually*,* you know? I have to think every day and I have to consider different things all the time. And I also learn a lot*,* basically every day. And that really challenges me*,* and I like that. I’ve also noticed that I can remember a lot more now.“ (P13)*.

Besides promoting cognition, respondents also valued the enhancement of motor skills to counteract MS-related deterioration of abilities. Another occupational resource mentioned was the structure provided by a regular routine of a day of work:

*“Having a routine is also good. You know*,* to have fixed times when you get up*,* when you show up at work or when you’re done. I think structure is really important for me as a person with MS. I need some kind of structured routine that I can follow to get me through the day.” (P10)*.

As a positive effect participants stated that work distracted them from focusing on their MS, evident in less perceived symptoms like tingling or numbness. Some participants assumed their condition might be worse if they would not work at all. Some also reported that the structured routine motivated them to focus more on a healthy lifestyle during their free time, such as diet and exercise. By choosing an active form of commuting (e.g., walking or cycling to work), some respondents reported already having increased their physical activity.

Several respondents particularly appreciated the option to work from home. Mentioned benefits included the omission of commuting to save time and sleep longer/get up later, a perceived better performance due to less interruptions by others as well as the general opportunity to tailor the workflow to individual needs, e.g., taking short breaks when feeling exhausted.

*“But at home*,* I actually work quite differently than I do in the office. Because I don’t have to get up so early. The commute is what makes the day so much longer. But when I’m working from home*,* I don’t have that. That means I can schedule my working day pretty flexibly. […] And to be honest*,* I usually get more done at home because there are fewer interruptions.” (P4)*.

In general, participants appreciated flexible working hours. Participants in shift work valued being able to choose shifts according to individual preferences and abilities, such as opting for night shifts instead of early shifts (which were perceived as more stressful). They reported less fatigue when a preferred shift could be chosen. Some respondents positively highlighted that their employer allowed them to attend medical appointments during working hours, making it easier to manage the disease alongside work.

##### c) Workplace equipment

Participants appreciated an ergonomic work environment including adjustable office chairs and height-adjustable desks. Additionally, elevators were particularly helpful for those with walking impairments and air conditioning was beneficial for those with temperature sensitivity. Relaxation rooms and places of retreat like break rooms and changing rooms were especially valued in combination with a daybed to lay down for an instant or a shower to cool down in the summer. One respondent mentioned the company canteen as a valuable work resource because it offered healthy food, allowing him to follow his MS diet.

#### Social factors

##### a) Supervisor

Interviewees appreciated supervisors with whom they could talk openly about the disease, who supported them by offering help and guidance and who allowed flexibility (e.g., working from home, adapting the tasks according to capabilities). Some respondents reported that supervisors even encouraged them to advance professionally, notwithstanding the MS:

*“That’s why I talked to my boss about it very early on*,* because this leadership development program and the diagnosis were kind of happening at the same time. And I was already thinking*,* why am I even doing this right now? Should I just step back from it? […] But then*,* I had a conversation with my supervisor*,* who encouraged me again: ‘Why don’t you just go through with it*,* and then we’ll see what happens.’ So*,* I definitely had some concerns about the development of my career after the diagnosis. But I was relieved of them.” (P9)*.

##### b) Colleagues

A good relationship with colleagues was also described as valuable, evident in mutual support, appreciation and shared and enjoyable lunch breaks. Some respondents reported receiving special support from colleagues in handling the disease in daily work life. For example, colleagues were willing to take over tasks that the affected person found difficult or filled in during absences (e.g., medical appointments). Consideration seemed to be particularly appreciated when it was not perceived as intrusive.

In general, participants reported experiencing self-efficacy when receiving praise and recognition from others, especially against the background of their MS:

*“I have to say*,* it’s just nice when you come home from work and you know that you’ve accomplished something […] The feeling of being needed is really nice. And even with MS*,* you’re not completely pushed aside*,* and you just realize that you can still contribute something to the big picture.” (P10)*.

#### Person-related factors

##### a) Attitude towards work

Many respondents reported a positive attitude towards their work and emphasized that they enjoyed working because they naturally liked being busy. They reported being generally ambitious and disciplined and spoke of a general sense of gratitude for being able to work.

Some participants expressed that they had experienced personal growth since their MS diagnosis. Some indicated that the MS even motivated them to advance their careers as long as it is possible.:

*“I’m doing a lot of further training right now […]. I’m just collecting everything and piling it up*,* like a little treasure of gold that I’m holding on to” (P16)*.

Others reported that they had to develop a carefree attitude to make daily work life easier:

*“I’ve changed my attitude and now I just don’t give a damn in most cases. So*,* when someone tries to reprimand me or point out any mistakes*,* I just say: ‘You know what*,* I don’t care. I’m not letting it get to me*,* I’ve got bigger problems.” (P24)*.

##### b) Handling one’s own disease

Most respondents reported that they had learned to adapt their workday to the disease to prevent stress. Interviewees stated that they had to learn to be always well organized, e.g., by prioritizing tasks and planning the workday according to levels of productivity. Additionally, they had to learn and respect their stress limits:

*“But actually*,* the biggest adjustment is that I pay more attention to myself and […] I try to adjust things more. I plan for the long term*,* organize myself better*,* […] and I also adjust my own expectations sometimes. I think if I didn’t have the disease*,* I might be even more ambitious.” (P16)*.

Reported strategies and possibilities to counter overload were regular vacations, especially after strenuous work periods, calling in sick when they had to without feeling guilty, an adequate number of breaks, making use of legal benefits (e.g., protection against dismissal, having a confirmed degree of disability) or choosing easier tasks for the day when they were no longer able to perform demanding activities. Some used the higher level of concentration in the morning to complete more complex work tasks, thus planning the day around the impairment. Some decided to always double-check the results of their work, others decided to take tasks home to calmly acquire knowledge needed at work because they realized that they need longer to learn new things.

#### Key consequences of work resources

Participants reported positive effects on cognition due to the opportunity to continuously learn new things, and on motor function through ongoing practice. The routine of work helped structure daily life and helped shift attention away from the disease. Overall, interviewees stated that they drew strength, motivation and energy from their work and appreciated the feeling of doing something useful.

## Discussion

### Summary of the main findings

Participants of our study reported different work stressors and resources and perceived effects on MS symptoms and work ability. Occupational stressors covered work demands (e.g., working overtime), organizational aspects (e.g., lack of options to work from home) and work equipment and environment (e.g., high temperatures at the workplace). Social work stressors occurred in the context of supervisors and employers (e.g., lack of understanding), colleagues (e.g., competitive pressure) and customers (e.g., conflicts). Person-related factors encompassed individual characteristics that reinforced stress (e.g., struggling to say no) and self-expectations (e.g., high-performance standards). Stressors were perceived to lead to e.g., fatigue, sensory disturbances or walking impairments.

Employees with MS often cope with work stress leading to physical MS symptoms, which results in the need for developing individual strategies (e.g., planning their day accordingly, doing some tasks at home). Occupational resources covered work content (e.g., diversified tasks), work organization (e.g., flexibility in organizing working hours and tasks) and workplace conditions (e.g., healthy canteen). Social work resources were described as support from understanding supervisors (e.g., praise and recognition, searching for common solutions) and colleagues (e.g., taking over tasks when needed). Person-related factors included a positive attitude towards work (e.g., feeling that work promotes health by enhancing cognition) and an expedient handling of the disease. This mainly included taking care of oneself by regularly taking breaks or utilizing legal benefits, e.g., additional vacation days. Resources were reported to make working days easier, enhance motivation and energy and improve skills (e.g., cognitive, fine motor). Participants emphasized the importance of work for their life satisfaction, as it distracts them from the disease and gives them a sense of being needed and making a valuable contribution to society.

### Comparison with prior research

Our findings regarding stressors and resources are in line with previous research in MS [2, 7, 10, 13, 15–18]. To the best of our knowledge, added value of our study to the research literature were the following additional effects of stressors and resources on MS symptoms: a perceived rise in sensory disturbances when the workload and periods of work stress increased, walking impairments when several people simultaneously approached ewMS with tasks or requests and positive effects of work tasks on cognition and fine motor skills. Other findings are in line with previous studies, e.g., stressful work days and mental effort that resulted in fatigue [20], a worsening of symptoms due to high temperatures at the workplace [14] or generally stressful work environments (e.g., schools, hospitals) leading to overload and cognitive challenges (i.e., becoming more forgetful, being more hesitant in conversations, having to think more than usually before speaking) [20].

Some participants in our study talked about impairments of fine motor skills or vision that made them question their capabilities of performing tasks, especially when impairments could put others or themselves in danger. This aspect was also identified by Coyne et al.: In that case, the fear of being a risk to others was mentioned as a reason to change, stop or reduce their work [21]. For ewMS, it could be useful to get in contact with healthcare providers, talk about potential fears and understand how the disease might affect their performance. Additionally, preparing for adjustments at the workplace as well as symptom management could be beneficial [21]. Other options to discuss potential resources and ways to get help, especially in the context of cognitive symptoms, can be support networks or groups [18].

A previous study found that support from work and social support contribute most strongly to health-related quality of life in women with MS who are still working [42]. These aspects were also mentioned as resources in our study but are not always available. In this context, participants also talked about negative aspects, such as competitive behavior, pressure, not being taken seriously, the need to justify themselves or patronizing behavior. For colleagues, it might be difficult to find a balance between providing help and support while not being overbearing at the same time. Yorkston et al. already identified similar factors, that is the desire of ewMS to decide for themselves, to be in control and the irritation if others worry too much and assume that workload is too much, which was also perceived as patronizing [20]. Overall, it seems important that colleagues as well as supervisors show interest in the MS, while leaving ewMS enough scope of action to make self-determined decisions. Communication and exchange are key factors for beneficial, social interactions. Yet, open communication is only possible if the MS is disclosed at the workplace. Education about and awareness of the disease for colleagues and especially supervisors is thus also important, and more education might also lead to more people affected talking openly about their MS.

Similar to the results of our study, Bogenschutz et al. identified potential fears related to future uncertainty (e.g., fear of the progression of the MS, fear of reduced work capacity) and related effects on the job choice (e.g., not applying for the dream job because it required too much traveling) [16]. In contrast to that, some participants in our study mentioned that the MS diagnosis gave them additional resilience and motivation to pursue their preferred careers and do further training. The more negative and skeptical perspectives in the study by Bogenschutz et al. might be due to the different study design: Even though the study also followed a qualitative approach, focus groups were conducted [16]. This might have led to an overrepresentation of negatives aspects due to the possibility that direct and outspoken participants can control these discussions [43].

Other potential reasons for more positive perspectives in our results could also be related to the concept of post-traumatic growth leading to an increase in functioning and capabilities after a decisive negative events: The results of a longitudinal study by Gil-Gonzáles et al. suggest significant positive consequences, such as an increase in personal strength and appreciation of life, for patients with MS over a follow-up of 36 months [44].

Yet, other motivational notions were also found in a qualitative study on the perspectives of women with MS (i.e., refusion to let the diagnosis define them and rather use it as an additional booster) [7]. These positive aspects might be especially beneficial for ewMS who recently received their diagnosis, who are not yet too restricted by the disease and are still able to work part-time or full-time. It could be useful to openly discuss options to advance in the career or pursue further education as long as the disease and individual circumstances permit it in order to counteract individual struggles (e.g., fears of the future, fear of losing the job) and thereby give additional strength and motivation to ewMS. This might also prevent fear of misjudgment on the part of employers and false assumption on the potential work ability of employees who were diagnosed with MS (and corresponding stressors as reported by, e.g. [18], and also found in our study).

While adaptations to the work tasks and awareness for symptoms [20] was also important for participants in our study, some participants in our study described that paying attention to your own body and to (mild/beginning) symptoms could also lead to a vicious circle (i.e., more focus on the symptoms leading to a perceived worsening of the condition and further distraction from work). In this case, relaxation or mindfulness exercises, e.g., meditation or mindfulness-based stress reduction, might be helpful to calm down and prevent additional stress [22].

Outside the context of work, a prior qualitative study from Germany found that individuals with MS sometimes perceived barriers when trying to implement dietary habits in their daily lives and talked about feelings of strength when referring to the daily impact of physical exercise [22]. In our study, some participants mentioned positive effects of a short way to work and a good canteen. Being able to combine the way to work with physical activity and having options for a healthy diet at the workplace could be effective and low-threshold options to stick to healthy habits.

### Limitations and strengths

A limitation of this study is that we had a highly educated sample with a rather short course of disease. Despite covering various sectors and industries, most of the participants pursued an academic career. As a result, the findings – with some exceptions – often relate to office-based work and may thus be limited and not generalizable. Yet, yielding generalizable findings is usually not a central feature of qualitative approaches [45]. Furthermore, we decided against member checking as we wanted to facilitate low threshold participation. Even though this might have added to the study’s rigor and allow for a justification on the part of the participants, member checking can have several disadvantages, such as the difficulty that participants might change their opinion or perspective after the interview, that they do not want to have ongoing contact with the researchers or a potentially low response rate [46]. Additionally, consistent with the healthy worker effect, we predominantly interviewed individuals (73% employed full-time) who were still fully capable of performing their jobs. According to the German MS Registry, 74% of patients are classified as having relapsing–remitting MS, whereas this proportion is higher in our sample at 85% [47]. Relapsing–remitting MS is typically associated with a slower progression of disability and longer periods of clinical stability. It is therefore conceivable that individuals in our sample may experience a lower symptom burden. This is relevant, as symptom severity is likely to shape both the perception of work-related stressors and resources and their impact on work ability. Individuals with fewer symptoms may perceive lower strain and be less adversely affected by workplace stressors. However, the selection of participants working at least 19 h per week was intentional, as our goal was to capture real-time reports of stressors and resources from individuals still engaged in their regular work routines in contrast to retrospective accounts (which are prone to bias) from those who had already left the workforce due to illness. Further research for Germany could include other professions and industries, e.g., blue-collar workers, and those with moderate and severe disability, who are likely to report different and a higher number of workplace factors jeopardizing their employment, as we already know from other studies (e.g [48]), . We successfully included both younger and older individuals as well as individuals with varying durations since their MS diagnosis. Most of our participants had relapsing-remitting MS, which is in line with the general distribution of MS-types (i.e., relapsing-remitting MS being the most prevalent phenotype [49]). As our findings might overrepresent views of individuals with this type of MS future research should include different forms of MS. Due to the qualitative study design, our findings are not generalizable in a statistical sense but provide context and insights into real-life experiences of a broad range of individuals affected. Another strength of our study is the inclusion of several researchers with different backgrounds, each with a distinct perspective on the data.

### Implications for research and practice

Our study showed that ewMS experience a variety of stressors that are perceived to negatively affect both their health and their ability to work. Some of these stressors can be mitigated through a supportive work environment and disability accommodations. Regarding the premises, ergonomic workplaces and elevators are particularly important in facilitating barrier-free work. Offering ewMS flexibility (i.e., flexible working hours, working from home) and making self-determined decisions possible enables them to better manage their condition, for example, by attending doctor’s appointments on workdays or by choosing adequate tasks with regard to their current well-being. These findings are consistent with the Job Demands–Resources (JD-R) model, which emphasizes that high job demands can lead to strain, whereas sufficient job resources (such as autonomy, social support, and appropriate equipment) can buffer negative effects and promote work engagement [12]. Quantitative research could investigate the relationship between identified stressors and symptoms, assessing measurable effect sizes to determine which stressors are particularly harmful. It would also be helpful to conduct longitudinal studies to track employment changes over time, thereby avoiding recall bias, i.e., distortions that arise when participants have to retrospectively reconstruct past events or experiences.

On an interpersonal level, educating supervisors and colleagues about MS might help to prevent prejudice and reduce stigmatization. A positive individual attitude towards employment despite MS could be fostered by information campaigns about working with MS (e.g., self-management strategies, information about legal benefits for ewMS). Supervisor training focusing on inclusive leadership and communication about chronic illness may further facilitate the implementation of accommodations and improve the work climate for ewMS. The impact of such measures should be scientifically evaluated and assessed for their potential effectiveness.

We also observed trends for occupation-specific patterns. For example, additional stressors emerged in healthcare settings (e.g., the responsible management of one’s own illness while providing patient care), whereas in manual trades resources were more limited (e.g., no possibility of working from home and restricted use of air conditioning due to fine dust exposure in woodworking). Future research should further investigate occupation-specific patterns across different employment types.

Although our findings are embedded in the context of Germany, the identified work-related stressors and resources are likely to be relevant across high-income countries. In countries with lower levels of social protection or less developed healthcare systems, ewMS may however experience an even greater burden from work-related stressors and may have reduced access to or utilization of legal benefits such as termination protection, occupational disability insurance, or additional vacation days, which were found to be an important work-related resource. German employees with disabilities benefit from a comparatively strong legal framework, including obligations for employers to provide suitable workplaces and far-reaching protections for severely disabled employees (e.g., workplace adaptations (§ 164 SGB IX), special leave (§ 208 SGB IX), protection against dismissal (§ 170 SGB IX) [50]. Similar principles of reasonable adjustment exist in other countries, such as the UK (Equality Act 2010) [51] and the USA (Americans with Disabilities Act) [52]. However, our findings suggest that ewMS may not always fully access or utilize these rights in practice, for example due to limited knowledge or fear of disclosure. Only half of our study participants had a formally registered degree of disability, which is required to access many of the related legal benefits. Awareness-raising appears to be beneficial in reducing prejudices among employers and improving employees’ knowledge of legal benefits.

## Conclusions

This study provides valuable insights into working with MS in Germany. Work stressors can be addressed through accessibility, flexibility in working hours, working tasks and location, as well as education for ewMS, colleagues, and supervisors. A reduction in stressors and increase in resources, particularly in flexibility, autonomy and self-determination might enable ewMS to remain productive and sustain long-term employment. This is particularly desirable considering that ewMS report improvements in their health due to work resources and working in general (i.e., social participation, contributing to society).

## Supplementary Information

Supplementary Material 1.

Supplementary Material 2.

Supplementary Material 3.

Supplementary Material 4.

## Data Availability

The datasets generated and/or analyzed during the current study are not publicly available due to confidentiality reasons, as the transcripts can contain sensitive information, but are available from the corresponding author on reasonable request if legal frameworks are not violated and responsibilities as well as confidentiality are clarified.

## Abbreviations

MS: Multiple sclerosis
ewMS: Employees with MS
RQ: Research questions
COREQ: Consolidated Criteria for Reporting Qualitative Research
SD: Standard deviation
