# Supplementary material for: Exploring work stressors and resources and their perceived effects on symptoms and work ability in employees with multiple sclerosis: a qualitative study
Source: BMC Neurol. 2026 Sep 24;26:607. doi: 10.1186/s12883-026-05417-z (PMC13602774; doi:10.1186/s12883-026-05417-z)
Supplement: Supplementary file 2 [file 12883_2026_5417_MOESM2_ESM.docx]

Additional File 3

**Interview guide**

**Opening question**

1. How are you currently feeling?

**Typical (work) day**

2. What does your typical workday look like?
   a. Could you please describe a typical workday?
   b. Could you please describe your tasks at work?
3. How does your MS (Multiple Sclerosis) manifest in your daily life?
4. What thoughts come to mind regarding the topic of "work and MS"?

**Stressors and resources at work**

5. Are there any factors at work that cause you stress? (e.g., working conditions, psychosocial stressors, work environment)
   a. Why do these factors feel burdensome to you?
   b. How do these factors affect you?

**Connection between stressors, symptoms, and work performance**

6. To what extent do you think the factors you just mentioned influence your MS?
   a. How do you think these factors affect the symptoms of your MS?
   b. How do you think these factors impact your performance or work ability?
   c. To what extent do you believe your work affects the progression of your condition?
   d. Is there anything else that comes to mind on this topic?

**Resources at work**

7. What do you like about your job?
   a. Are there any factors that benefit you?
   b. Why do these factors benefit you?

**Connection between resources, symptoms and work performance**

8. To what extent do you believe the factors you just mentioned affect your MS disease?
   a. How do you think these factors affect the symptoms of your MS?
   b. How do you think these factors impact your performance or work ability?
   c. To what extent do you believe your work affects the progression of your condition?
   d. Is there anything else that comes to mind on this topic?

**Dealing with MS at the workplace**

9. To what extent is your workplace informed about your condition?
   a. Who is informed?
   b. What were the reasons for or against disclosing your condition?
10. Would you like to see changes in your workplace or work situation due to your MS?
    a. Why would these changes help you?
11. Have you made any adjustments to your workplace or work situation due to your MS?
12. Has your employer made any adjustments to your workplace or work situation as a result of your MS? (if disclosed)

**Professional career and MS**

13. Could you please share more about your professional career so far?
14. To what extent do you believe your MS has influenced your professional career?

**Closing questions**

15. What does your current treatment look like, and what medications are you taking?
16. Are there other responsibilities in your daily life beyond work, such as childcare or caregiving for relatives?
17. How did you hear about our study?
18. That concludes my questions. Is there anything else you would like to share regarding this topic that has not yet been addressed?
