# Supplementary material for: Exploring work stressors and resources and their perceived effects on symptoms and work ability in employees with multiple sclerosis: a qualitative study
Source: BMC Neurol. 2026 Sep 24;26:607. doi: 10.1186/s12883-026-05417-z (PMC13602774; doi:10.1186/s12883-026-05417-z)
Supplement: Supplementary file 3 [file 12883_2026_5417_MOESM3_ESM.docx]

Additional File 2

**Background questionnaire**

Dear Participant,

Please take 5–10 minutes to complete the following questionnaire before your interview. For each question, select the answer that you feel best applies to you.

Before filling out the questionnaire, please carefully read the study information and the data protection details. Your data can only be used if you confirm the consent form by checking the box.

Thank you very much!

**1. How old are you?** ____

**2. Which gender do you identify with?**

☐ Female	☐ Male	☐ Diverse

**3. What is your highest educational qualification?**

☐ No school-leaving certificate

☐ German ‘Haupt-/Volksschulabschluss’ (i.e., lower secondary school-leaving certificate)

☐ German ‘Mittlere Reife/Realschulabschluss‘ (i.e., intermediate secondary school-leaving certificate)

☐ German ‘Fachhochschulreife’ (i.e., advanced technical college entrance qualification)

☐ German ‘Abitur/allgemeine Hochschulreife‘ (i.e., general qualification for university entrance)

☐ University education (bachelor’s/master’s degree or higher)

**4. What is your current profession**? _______________________________

**5. Do you work...?**

☐ Full-time	☐ Part-time with ____ hours per week

**6. When was your MS diagnosed?**

☐ Within the last 12 months

☐ ____ years ago

7. **What is your current disease course?**

☐ Clinically isolated syndrome (CIS)

☐ Relapsing-remitting MS (RRMS)

☐ Secondary progressive MS (SPMS)

☐ Primary progressive MS (PPMS)

☐ Prefer not to disclose

**8. If you have a relapsing disease course, when did you have your last relapse?**

____ months ago

**9. How many relapses have you experienced since the onset of the disease?** ____

**10. How far can you currently walk on level ground?**

☐ < 10 m

☐ 10–500 m

☐ 500 m

**11. Are you currently using a mobility aid?**

☐ No mobility aid

☐ Cane

☐ Walker (Rollator)

☐ Wheelchair

☐ Other: ____

**12. Has a degree of disability been determined for you?**

☐ No

☐ Yes, with the following degree of disability: ____

**13.** **Are you currently suffering from any of the following conditions, independent of your MS?**

☐ Cardiovascular diseases (e.g., heart attack, heart failure)

☐ Respiratory diseases (e.g., lung cancer, asthma, bronchitis)

☐ Gastrointestinal diseases

☐ Diseases of the liver, gallbladder, or pancreas

☐ Diseases of the kidneys and urinary tract

☐ Metabolic diseases (e.g., diabetes, gout)

☐ Hormonal diseases (e.g., thyroid disorders)

☐ Spinal diseases (e.g., herniated disc)

☐ Bone and joint diseases

☐ Neurological diseases (e.g., stroke, epilepsy)

☐ Mental illnesses (e.g., depression, anxiety)

☐ Addiction disorders

☐ Infectious diseases (e.g., tuberculosis)

☐ Other conditions, specifically: ____

**14. If you have checked one or more conditions, you have the opportunity to add a comment here:**

______________________________________________________________________________________________________________________________________________________________________________________________________________________________
