# Supplementary material for: Exploring work stressors and resources and their perceived effects on symptoms and work ability in employees with multiple sclerosis: a qualitative study
Source: BMC Neurol. 2026 Sep 24;26:607. doi: 10.1186/s12883-026-05417-z (PMC13602774; doi:10.1186/s12883-026-05417-z)
Supplement: Supplementary file 4 [file 12883_2026_5417_MOESM4_ESM.docx]

Additional File 4

**Occupational information of the participants**

**Table S1. Occupational information of the participants.**

| Participant | Occupation | Employed full-time | Part-time working hours |
| --- | --- | --- | --- |
| P1 | Social worker in daily assistance and activation | no | 25 |
| P2 | Doctoral candidate | yes |  |
| P3 | Medical assistent | yes |  |
| P4 | Software developer | yes |  |
| P5 | Food quality assurance specialist | yes |  |
| P6 | Pediatric nurse | no | 35 |
| P7 | Team leader in biotechnology | yes |  |
| P8 | Marketing clerk | yes |  |
| P9 | Team leader in IT department | yes |  |
| P10 | Event technology specialist | no | 30 |
| P11 | Carpenter | yes |  |
| P12 | Team leader in upper-level public services | yes |  |
| P13 | Rehabilitation technician | yes |  |
| P14 | Network and project manager | yes |  |
| P15 | Medical writer & psychological counselor | yes |  |
| P16 | Research associate | yes |  |
| P17 | Teacher & freelancer | no | 30 |
| P18 | Biologist & doctoral candidate | yes |  |
| P19 | Nurse | no | 30 |
| P20 | Risk manager for construction-related risks | no | 25 |
| P21 | Social worker in resident.child and youth services | no | 31,75 |
| P22 | Parish assistant | yes |  |
| P23 | Legal trainee | yes |  |
| P24 | Administrative clerk | yes |  |
| P25 | Dentist | yes |  |
| P26 | Head of human resources | yes |  |
